# Oxygen as a Remedial Agent

**Published:** 1873-01

**Authors:** J. Henry Davenport


### SELECTED ARTICLES.

---

### ARTICLE V.

### *Oxygen as a Remedial Agent.*

BY J. HENRY DAVENPORT, M. D.

The idea of inhaling oxygen as a remedial agent is by no means a new one. It was practiced sixty or seventy years ago by Dr. Beddoes, but given up because the gas could not then be obtained in sufficient quantity, cheapness and purity. It was thought that the impurities caused irritation of the lungs, and even salivation had resulted where the oxygen had been procured from the red oxide of mercury. Moreover, caoutchouc in its present form was then unknown to the arts, and there existed a difficulty in handling the gas which the rubber bag of to-day has entirely removed. Dr. Beddoes used small closets and chambers, which were filled with oxygen at large expense. But it was the discovery by Tessie du Motay of a process of obtaining oxygen from the atmosphere in almost absolute purity, the only adulteration being the admixture of a slight proportion of nitrogen, a normal element of the atmosphere, that at once removed the objections of impurity and high cost. This process is so admirable for its simplicity and perfection that, like most great advances in science, the wonder is that it was not discovered before. His method is as follows: Manganate of soda is heated to a very high temperature in iron retorts, and while in this molten state a current of atmospheric air is made to pass over it. The salt vividly absorbs the oxygen of the air in large quantities, together with a little of the nitrogen. The current of air is then shut off, and one of superheated steam substituted. The oxygen absorbed from the air is given up to the steam, and with it passes along to a condenser, where the steam is condensed, and whence the oxygen is conducted away to a gasometer. The sole expense is that of the fuel, as the manganate of soda can be used repeatedly. Oxygen thus obtained has found many uses in the arts; and in New York most especially in cheaply furnishing the blast for the lime light for night illuminations of theatres, street advertisements, &c. It is even proposed by the N. Y. Oxygen Gas Company to illumine the principal streets of the city by substituting the lime light for ordinary street lamps, and it is claimed that oxygen can be furnished as cheaply as the coal gas.* Motay was not thinking so much of medicine as of more remunerative arts when he published his process. Nor was it until May 1st, 1870, that a small number of the most eminent physicians of New York, "believing that a benefit to the public would result from having a sufficient supply oxygen, in a portable form, for medical use at all times accessible," suggested to the Oxygen Gas Company the establishment of a depot for that purpose in a central locality. Among the signers were George T. Elliott, Wm. H. Van Buren, Alonzo Clarke, Willard Parker, Austin Flint, and other names of equal fame in the medical world. Accordingly an office was opened on Broadway, near the Fifth

*Since the above, the oxhydric light has been tried in Paris in the street lamps of the Boulevard des Italiens. It was not a success, and is now discontinued there on account of its complicated management and its expense.

Avenue Hotel. The gas is furnished in copper or iron cylinders, in shape and size somewhat resembling the familiar chemical fire extinguishers, and containing one or two hundred gallons of oxygen compressed by a force equal to seventeen atmospheres. From one of these as much is drawn off into a rubber bag as is needed from time to time for inhalation. During the second year that oxygen was thus offered in portable form to the medical public of New York, upwards of 400,000 gallons of the gas were sold for medical purposes, and the demand is constantly increasing.

A new field was thus thrown open to competition. During the same year (1870) the prize of the Alumni Association of the College of Physicians and Surgeons was awarded to Dr. A. J. Smith, now Lecturer on Materia Medica in the Long Island College Hospital, for an essay on "Oxygen as a Remedy in Disease." The essay follows the history of the gas from its discovery during the last century, by Dr. Priestly, to its dispensation in New York in 1870. Dr. Priestly was the discoverer, not only of oxygen, but also of its peculiar relation to animal life, and suggested its possible employment by the physicians. But his words fell on ears as unwilling as those of the surgeons to whom Sir Humphrey Davy formerly recommended the possible use of nitrous oxide as an anæsthetic.

Dr. Smith's essay having brought the subject prominently before the profession, there appeared during the succeeding year various articles in the local journals of New York, speaking highly of the power of oxygen, and from a study of the cases thus made public the following therapeutical uses may be deduced.

The diseases in which oxygen is of benefit are conveniently divided into three classes:

First: Those involving dyspnœa, which are essentially acute. The oxygen here acts only as a palliative, serving to bridge over a chasm, and give nature an opportunity to recover her scattered forces. It merely prevents death from absolute suffocation arising from imperfect aeration, or, more properly speaking, oxygenation of the blood. Examples of this class are the acute stages of bronchitis, Bright's disease, heart disease, uræmia, opium poisoning, and the like.

Second: Those involving defective nutrition or excretion, which are essentially chronic. Oxygen here acts as a tonic, increasing the weight and strength, and visibly restoring the natural ruddy hue of the face in health. It has in this way been found valuable in phthisis, emphysema, acute dyspepsia, anæmia, &c. In these affections, three or four gallons are inhaled daily.

Third: In certain spasmodic diseases, oxygen has an unexpected but welcome effect. It is a sedative, though an indirect one, and has been found of great use in asthma, chorea, epilepsy, and many other ailments of a convulsive nature.

Perhaps the mode of action of oxygen can be best appreciated from the recital of a few cases from the journals.

A child, 2½ years old, was at the point of death from bronchitis intercurrent with measles. Dr. Little was called in consultation, and considered the case hopeless. The respiration was 80 per minute, accompanied by rales audible at some distance from the bed; pulse too frequent to be counted; face pale and dusky; extremities cool; body excessively hot. About four gallons of oxygen were administered at 10 P. M. with little effect. At midnight the administration was resumed, and continued without intermission until 3 A. M. Within an hour the respiration had fallen to 60, and the pulse to about 160. By 2 A. M. the face had regained its natural color, the mucous rales were no longer audible unless the ear were placed to the chest. The inhalation was continued irregularly for five hours longer, when it was wholly discontinued. The next day convalescence was fully established.

Dr. Peaslee related, before the New York Medical Journal Association, a case that came under his notice of an aged lady who had been suffering from disease of the heart for some two years. A sudden attack of dyspnœa came on. About five gallons of oxygen was inhaled every ten minutes. Very soon the dyspnœa was relieved, the lividity of the lips removed, and she was comparatively comfortable. Moreover, the oxygen aroused her from the state of stupor into which she had sunk, and to which she would regularly return on stoppage of the oxygen. There was, of course, no arrest of the pulmonary œdema. The liquid rose higher and higher, the patient sitting erect. In Dr. Peaslee's opinion, the gas did great service in unquestionably prolonging her life until the medicine given for the œdema had time to operate, which it did in the course of about half a day. The next day there was a relapse. Oxygen was again resorted to. Matters continued thus for about ten days, and the patient seemed to be out of all danger for the time being. But symptoms of double pneumonia setting in, she died very soon. After the outbreak of pneumonia, oxygen was given for three days, and enabled her to converse with her family up to ten minutes before her death. In this case the patient knew her condition perfectly, and desired to live until her son and daughter could come on from the West; and Dr. P. is convinced that the oxygen alone enabled her to accomplish her desire.

The case of the late Secretary Stanton, as related by Dr. Smith, was one in which oxygen was the means of averting a great deal of suffering. He was subject to severe paroxysms of asthma, which seriously aggravated his condition during his last illness. His physician, Surgeon General Barnes, U. S. A., procured a supply of compressed oxygen, of which three or four cubic feet were inhaled daily. It controlled the paroxysms completely, and, so far as the asthma was concerned, met every indication. Gen. Barnes expressed himself warmly in favor of the gas in similar cases, and stated that both he and his illustrious patient were entirely satisfied with its effects.

We cannot, for want of space, rehearse any cases illustrating the tonic effect of oxygen. Demarquay even asserts its remedial power over some forms of diabetes, and states that by its agency he has reduced the sugar in the urine by one-half, the diet remaining unchanged.

Among the most interesting clinical observations have been those of Dr. Smith on the effect of inhalations of oxygen upon the pulse. The first series of 102 cases were upon 11 phthisical patients—in 62 of whom the pulse was retarded 10 beats per minute; in 16 the rate was not modified, and in 12 there was an increase of 6 beats. Among the 11 patients experimented upon, 3 uniformly presented a lowering of the pulse, while the 8 others varied, though usually the pulse was diminished.

In a second series of 12 observations on 12 healthy individuals, 4 presented no modification, while the 8 others presented a diminution of 9 beats per minute.

If the reduction of the pulse had been confined to the phthisical cases, we might suppose that the action of the oxygen had been merely that of a stimulant, such as brandy; but when we consider the effect produced on the system in health, this view, Dr. Smith thinks, is not tenable. The indication seems to be that oxygen is an arterial sedative, not perhaps analogous to digitalis or veratrum viride, but acting indirectly, causing a change in the blood, which facilitates the circulation and lessens the labor of the heart. This sedative action was sufficient, in a case of nervous debility related by Dr. Peaslee, to cause sleep where chloral had only a partial effect.

A third series of experiments was made with the aid of the sphygmograph. It is impossible here in the absence of plates to dwell on the results obtained. But they are worthy of the attention of those versed in the study of sphygmography. In a general way, we may say that the height of the curve is increased and the dicrotism more pronounced. Oxygen, in a word, gives greater regularity to the pulse.

The amount of oxygen used in these experiments was generally about eight gallons, which was inhaled gradually in the course of a few minutes.

The method of action of oxygen in relieving dyspnœa is sufficiently obvious. If the patient breathes an atmosphere containing an increased proportion of oxygen, the blood is more quickly and thoroughly saturated, and the breathing made proportionally easier. But, although it may be said in general terms that nearly every disease involving dyspnœa may be at least temporarily relieved by inhaling oxygen, still this help is not always so speedily found. Though so clearly indicated theoretically, we often meet with rugged obstacles in practice. Nor is it by any means clear why a remedy so magical at some times is so powerless at others. At present, several reasons have been offered in explanation. The blood is said to be very nearly saturated with oxygen in ordinary respiration, and even an atmosphere highly charged with the gas cannot add very greatly to the quantity absorbed. If there be a partial obstruction in the air-passages, leaving the air-cells intact—a condition, for example, which obtains in bronchitis—then, by adding to the little air that really is inspired an extra amount of oxygen, we may preserve the effect of the standard respiration.

But if the air-cells of a large portion of the lung are filled up, a condition that is found in extensive pneumonia, add as much oxygen as we please to the air which penetrates the remainder of available lung, and yet we may not be able to force into the system the usual and necessary supply.

Moreover, as Dr. Neftel observes, we must remember that the process of oxygenation is subject indirectly to the nervous system through its connection with respiration, which, with its complex and ingenious co-ordination of movements, is governed by the respiratory centre in the medulla oblongata. Whenever, then, asphyxia is due not to want of air in the lungs, but to a diseased condition of the nervous system, we cannot expect much from the use of oxygen. Again, since the red corpuscles are the chief agents in the reception of oxygen, it is evident that when they are deficient, either in number, as in leukæmia, or in quality, as in diabetes, oxygen must often fail to be of any marked benefit.

It would be interesting to detail some of the physiological experiments with oxygen on animals. We have often noted when breathing oxygen the ease with which the breath can be held for a period almost impossible when inhaling merely atmospheric air. By saturating the system with oxygen, we obtain a condition of "apnæa," in which respiration practically ceases for the time being. It has even been shown that an animal in the convulsions caused by strychnia has complete relief from them while in this condition of "apnœa." Some experiments of our own in this matter are reserved for a future paper.—*Boston Medical and Surgical Journal.*

---

